# Small organic molecule disruptors of Cav3.2 - USP5 interactions reverse inflammatory and neuropathic pain

**DOI:** 10.1186/s12990-015-0011-8

**Published:** 2015-03-14

**Authors:** Vinicius M Gadotti, Agustin Garcia Caballero, N Daniel Berger, Clare M Gladding, Lina Chen, Tom A Pfeifer, Gerald W Zamponi

**Affiliations:** Department of Physiology and Pharmacology, Cumming School of Medicine, University of Calgary, 3330 Hospital Drive, NW Calgary, T2N 4N1 Canada; Centre for Drug Research and Development, 2405 Wesbrook Mall – 4th Floor, Vancouver, BC V6T 1Z3 Canada

**Keywords:** T-type channels, USP5, Chronic pain, Suramin, Gossypetin

## Abstract

**Background:**

Cav3.2 channels facilitate nociceptive transmission and are upregulated in DRG neurons in response to nerve injury or peripheral inflammation. We reported that this enhancement of Cav3.2 currents in afferent neurons is mediated by deubiquitination of the channels by the deubiquitinase USP5, and that disrupting USP5/Cav3.2 channel interactions protected from inflammatory and neuropathic pain.

**Results:**

Here we describe the development of a small molecule screening assay for USP5-Cav3.2 disruptors, and report on two hits of a ~5000 compound screen - suramin and the flavonoid gossypetin. In mouse models of inflammatory pain and neuropathic pain, both suramin and gossypetin produced dose-dependent and long-lasting mechanical anti-hyperalgesia that was abolished or greatly attenuated in Cav3.2 null mice. Suramin and Cav3.2/USP5 Tat-disruptor peptides were also tested in models of diabetic neuropathy and visceral pain, and provided remarkable protection.

**Conclusions:**

Overall, our findings provide proof of concept for a new class of analgesics that target T-type channel deubiquitination.

**Electronic supplementary material:**

The online version of this article (doi:10.1186/s12990-015-0011-8) contains supplementary material, which is available to authorized users.

## Background

Chronic pain is a major burden on society and despite increased understanding of the neurobiology of pain, only a few novel classes of analgesic compounds have entered the clinic. Thus the identification of new analgesics is of critical necessity. In the primary afferent pain pathway, voltage gated T-type calcium channels sustain neuronal firing and appear to contribute to neurotransmitter release at afferent terminals in the spinal dorsal horn [1,2]. This increased neuronal excitability leads to amplification of sensory transmission resulting in pathological pain perception [3]. Therefore, blocking T-type channels is known to mediate analgesia. For example, systemic delivery of the poorly selective T-type channel inhibitors mibefradil and ethosuximide produces antinociceptive effects in rodents [4,5]. More recently developed compounds with better selectivity for T-type channels such as TTA-A2 [6], TTA-P2 [7], M12 [8] and N12 [9] are also protective in various models of inflammatory and neuropathic pain. The prominent T-type channel subtype expressed in peripheral afferents is Cav3.2, and consequently selective knockdown of the Cav3.2 mRNA via intrathecal delivery of antisense oligonucleotides produces antinociceptive and anti-hyperalgesic effects [10]. On the other hand, nerve injury or peripheral inflammation result in upregulation of T-type calcium channels in primary afferent fibers, which in turn contributes to pain hypersensitivity [11-14]. Our group has recently identified a new cellular pathway that contributes to this aberrant upregulation of Cav3.2 channels in chronic pain states [15]. We showed that Cav3.2 channels associate with USP5, a deubiquitinating enzyme [16,17] that is upregulated in dorsal root ganglion (DRG) and dorsal horn tissue in response to either nerve injury or peripheral inflammation. This then leads to enhanced deubiquitination of Cav3.2 channels and consequently an increase in Cav3.2 channel density in the plasma membrane at cell bodies and likely at nerve terminals. Knockdown of USP5 via shRNA or preventing its association with the channel by using Tat epitope fused disruptor peptides decreased Cav3.2 protein levels and produced analgesia in models of inflammatory and neuropathic pain in mice. The major benefit of this approach over conventional T-type channel inhibitors is that it targets aberrant upregulation of the channels, while sparing normal channel function.

Here, we describe the development of an ELISA-based assay to screen a library of pharmacologically active compounds (including clinically used drugs) for molecules capable of disrupting the USP5-Cav3.2 interaction. Two of the identified hits, suramin and gossypetin, were found to be active in various mouse models of inflammatory and neuropathic pain, while their activities were attenuated or lost in Cav3.2 null mice. Hence, our findings validate small compound inhibitors of Cav3.2 deubiquitination as a new strategy for treating chronic pain.

## Results

### Blocking Cav3.2 channel deubiquitination protects from visceral and diabetic pain

**Figure 1:**
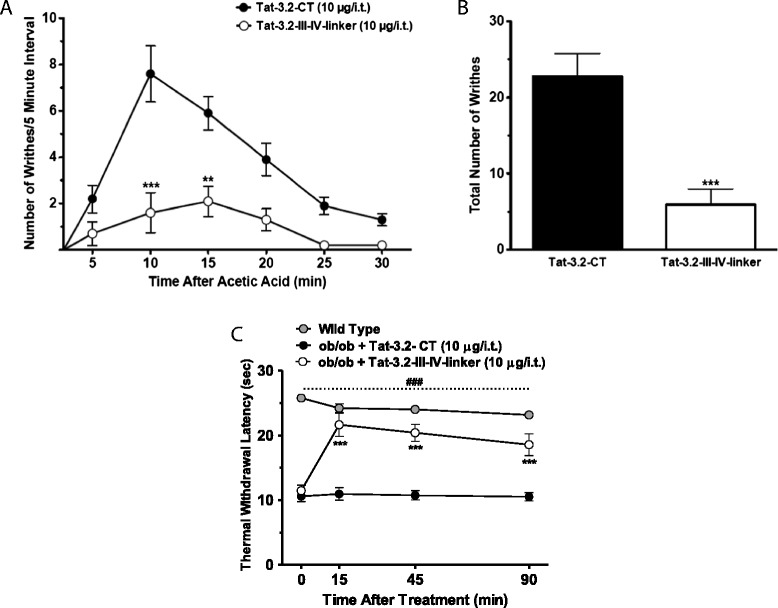
**Effects of the Tat-Cav3.2-III-IV linker peptide in mouse models of visceral pain and diabetic neuropathy. (A)** Blind analyses of writhing responses of mice treated with the Tat-3.2-III-IV linker (10.0 μg/i.t.) or the Tat-3.2-CT (control, 10.0 μg/i.t.) peptides 1 hour prior to acetic acid injections. **(B)** total number of writhing responses of acetic acid injected mice treated with Tat-3.2-III-IV linker or the Tat-3.2-CT peptides. **(C)** Time course of antihyperalgesic effect of the Tat-3.2-III-IV linker (10.0 μg/i.t.) or the Tat-3.2-CT (10.0 μg/i.t.) peptides delivered to diabetic neuropathic (*ob/ob*) and wild-type mice. The experimenter was blind to the treatment in panels **A**, **B** and **C**. Each circle/bar represents the mean ± S.E.M. (n=7 in panel C; and n=10 in panels **A** and **B**) and is representative of 2 independent sets of experiments. Statistical analyses were performed by two-way ANOVA followed by a Tukey’s test (panels **A** and **C**), and unpaired Student’s t-test (panel **B**). The dashed line and hashtag indicate the range of data points where diabetic neuropathic animals differed from the non-diabetic group (P<0.001).

We recently reported that Tat-epitope fused peptides corresponding to the USP5 interaction site on the Cav3.2 channel’s intracellular domain III-IV linker (Tat-Cav3.2-III-IV) protected mice from CFA-induced inflammatory pain, and from sciatic nerve ligation-induced neuropathic pain [15]. To determine if this mechanism was also active in other pain conditions, we tested the efficacy of the peptides in models of acute visceral pain and diabetic neuropathy. As shown in Figure 1A and B, intraperitoneal (i.p.) administration of acetic acid (0.9%) induced a writhing response in mice treated with a control Tat-peptide. In contrast, no such writhing behavior was observed in mice injected i.p. with vehicle (Additional file 1: Figure S1). Mice treated via intrathecal delivery of the Tat-Cav3.2-III-IV peptide one hour prior to the delivery of acetic acid displayed a robust dose-dependent reduction in the associated pain response in a blinded experiment. A positive control (Diacerein, 50 μg/i.t., 15 minutes prior acetic acid injection) similarly inhibited pain responses (75 ± 3%, Additional file 1: Figure S1).

Next we tested the same peptide in in chronically obese (*ob/ob*) mice as a model of diabetic neuropathy. These mice develop diabetes and an associated polyneuropathy that gives rise to increased thermal and mechanical hypersensitivity compared to non-diabetic mice. As shown in Figure 1C, intrathecal delivery of the Tat-Cav3.2-III-IV peptide, but not the control Tat-3.2-CT peptide, mediated a time-dependent inhibition of thermal hypersensitivity. Altogether, these data show that the USP5-mediated dysregulation of Cav3.2 channels occurs during both diabetic neuropathic pain and during chemically-induced visceral pain, and disrupting this process mediates analgesia.

### Development of a Cav3.2-USP5 ELISA assay

**Figure 2:**
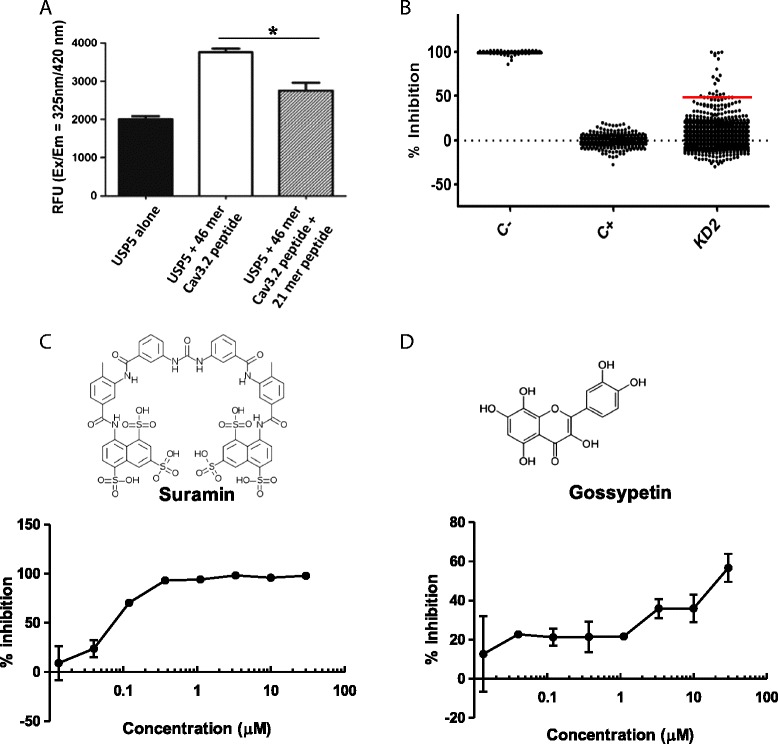
**Cav3.2/USP5 ELISA screening results. (A)** Competition affinity ELISA assay. A non-biotinylated human Cav3.2_1569–1589_-III-IV peptide (21 mer) was used to compete off the interaction between the biotinylated Cav3.2 Cav3.2_1556–1602_-III-IV peptide (46 mer) and hUSP5. **(B)** Screening of a 5000 bioactive compound library using a 384 well capacity ELISA. A group of active compounds (~50% inhibition) were identified. **(C)** Structure and concentration response curve for suramin in the ELISA assay. **(D)** Structure and concentration response curve for gossypetin in the ELISA assay.

While our Tat peptide approach delivered proof of concept for targeting the Cav3.2-USP5 interaction as a strategy for targeting various pain conditions, small organic mimetics of these peptides are a preferred strategy for therapeutics. We therefore developed an ELISA-based assay for identifying small organic disruptors of the Cav3.2-USP5 interaction. We used a 46 meric HPLC purified synthetic peptide corresponding to the human Cav3.2 III-IV cytosolic linker and a purified recombinant human USP5 (long splice isoform) as the basis for the assay. A biotinylated version of the 46 mer was conjugated to a neutravidin-coated plate and recombinant human USP5 was added. To detect USP5 bound to this immobilized Cav3.2 III-IV linker peptide we used a rabbit polyclonal anti-USP5 antibody, followed by the addition of an HRP-conjugated secondary antibody. We then used a QuantaBlu substrate to read the relative fluorescent units (RFU) at excitation and emission wavelengths of 325 nm/440 nm. To ensure disruption of this interaction was possible we repeated the assay in the presence of a non-biotinylated Cav3.2 III-IV peptide (21 mer) as a competitor. USP5 robustly bound to the biotinylated 46 mer Cav3.2-III-IV linker peptide, and this interaction was reduced by > 50% in the presence of the peptide competitor (Figure 2A) thus validating the assay. The incomplete competition observed with the non-biotinylated peptide is likely due to its shorter amino acid composition (21 mer) when compared with the biotinylated peptide longer version (46 mer).

### Screening for modulators of the USP5-Cav3.2 interaction

**Figure 3:**
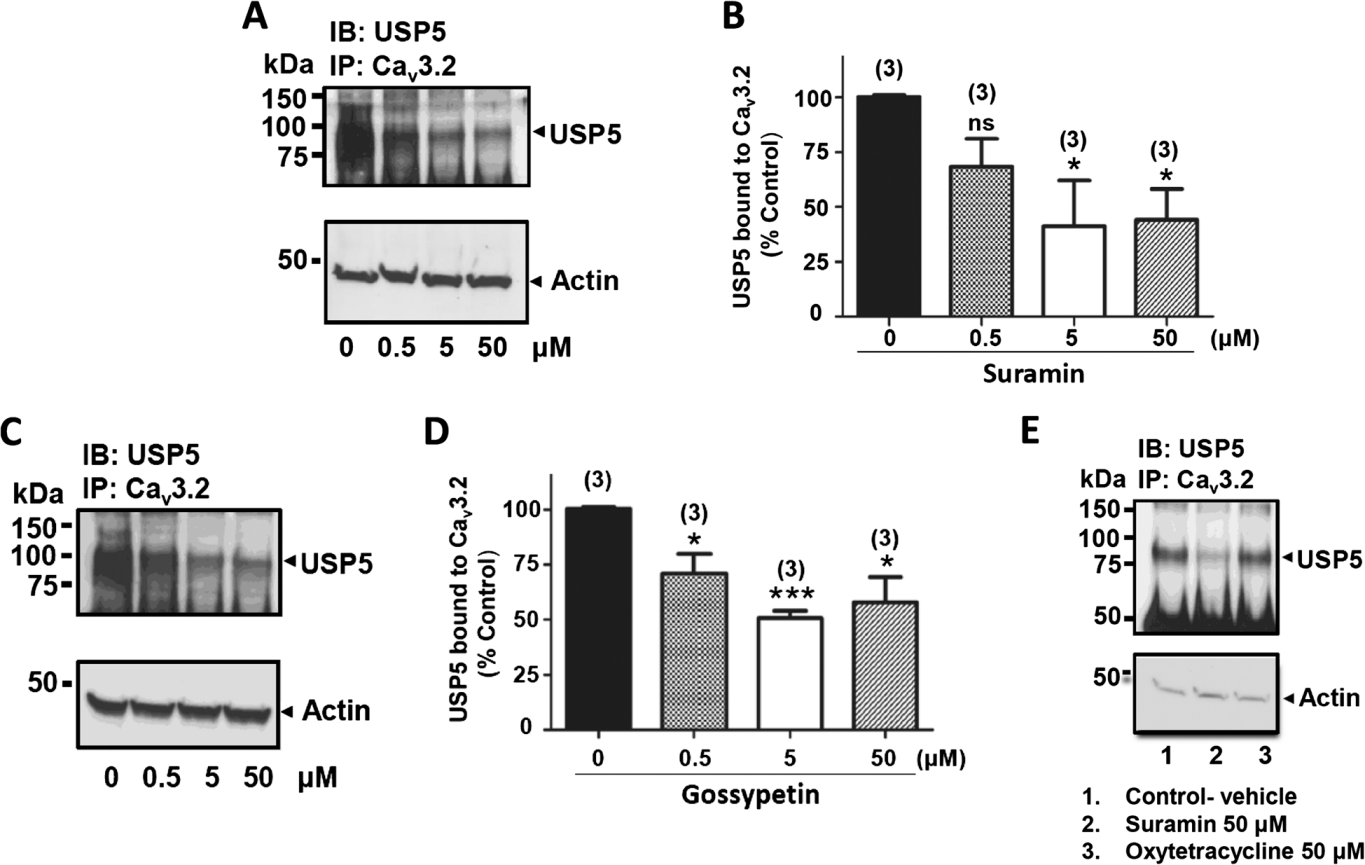
**Effect of suramin and gossypetin on USP5-Cav3.2 interactions. (A)** Cav3.2 immunoprecipitates incubated with different concentrations of suramin and probed for USP5 by Western blot. An actin loading control is shown. **(B)** Quantification of USP5 bound to Cav3.2 immunoprecipitates by densitometry. **(C)** Cav3.2 immunoprecipitates incubated with different concentrations of gossypetin and probed for USP5 by Western blot. An actin loading control is shown. **(D)** Quantification of USP5 bound to Cav3.2 immunoprecipitates by densitometry. **(E)** Cav3.2 immunoprecipitates incubated with vehicle (DMSO, lane 1), 50 μM suramin (lane 2) and 50 μM oxytetracycline (lane 3).

The ELISA assay was refined and miniaturized to a 384 well format, and used to screen a small library of (~5000) bioactive compounds (KD2). The resulting screen had a Z’ of 0.79 with a signal to noise of 162 (Figure 2B). A set of 18 compounds were above 50% inhibition of the Cav3.2/USP5 interaction which was the arbitrary cut-off selected for this screenEight compounds were confirmed with concentration-based activity from screening plate samples, with 6 being re-confirmed from freshly ordered powder. These six compounds were then subjected to a secondary screen involving co-immunoprecipitations between USP5 and Cav3.2 from whole mouse brain lysate. This led to the confirmation of two compounds, suramin and gossypetin whose concentration response in the ELISA assay are shown in Figures 2C and D, respectively. Suramin is a large molecular weight compound that contains multiple benzene rings and is used clinically to control helminth and protozoal infections [18]. It is commonly thought of as a purinergic receptor antagonist [19]. Gossypetin, on the other hand, is a flavonoid isolated from *Hibiscus sabdariffa.* It has antimicrobial activity, and is reported to have health benefits in conditions such as cardiovascular disease [20,21]. The concentration dependence of the effects of both compounds on the co-immunoprecipitation between Cav3.2 channels and USP5 are illustrated in Figure 3. Suramin and gossypetin inhibited USP5 binding to Cav3.2 channels by 50-60% at 5 μM (Figure 3A-D). In contrast, oxytetracycline hydrochloride was used as negative control as it lacked inhibitory properties in the ELISA (Figure 3E). Altogether, these data reveal suramin and gossypetin as potential small organic disruptors of USP5 interactions with Cav3.2.

### Effect of suramin and gossypetin on mechanical hyperalgesia produced by sciatic nerve injury

**Figure 4:**
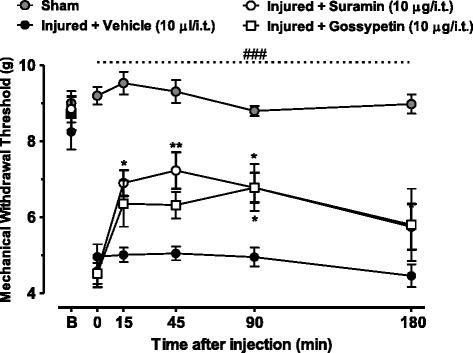
**Antihyperalgesic effect of compounds in a model of neuropathic pain.** Time course of the antihyperalgesic effect of intrathecally (i.t.) delivered suramin (10.0 μg/i.t.) or gossypetin (10.0 μg/i.t.) on mechanical hyperalgesia of mice subjected to nerve injury two weeks prior to the experiment. Each circle represents the mean ± S.E.M. (n=6-7) and is representative of 2 independent sets of experiments. Statistical analyses were performed by two-way ANOVA followed by a Tukey’s test. The dashed line and number symbol indicate the range of data points where injured animals differed from the sham group (P<0.001).

To establish if the compounds are also able to reduce mechanical hyperalgesia caused by peripheral nerve damage, we tested the effects of the compounds 14 days after partial sciatic nerve ligation. The sciatic nerve injury produced marked and long-lasting mechanical hyperalgesia with behavioral abnormalities that were evident for several weeks after the injury when compared with the baseline response and sham operated group (P< 0.001, Figure 4). When compared to the neuropathic control group, treatment of mice with suramin (10 μg/i.t.) or gossypetin (10 μg/i.t.), but not the vehicle (PBS, 10 μl/i.t.) produced rapid anti-hyperalgesia that remained significant up to 90 minutes after treatment (P<0.01, Figure 4).

### Effect of compounds on mechanical hyperalgesia induced by peripheral inflammation

**Figure 5:**
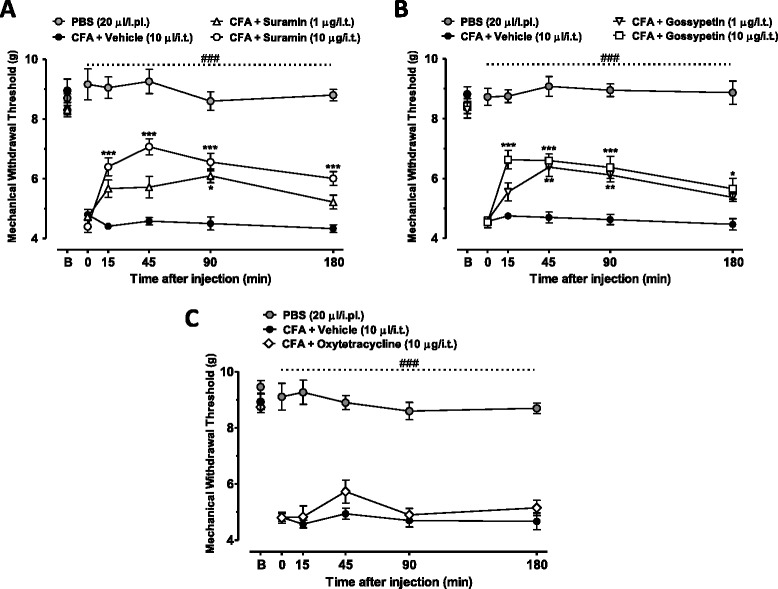
**Antihyperalgesic effect of compounds against persistent inflammatory pain.** Time course and dose-dependent antihyperalgesic effect of **(A)** suramin (1.0-10.0 μg/i.t.), **(B)** gossypetin (1.0-10.0 μg/i.t.) and **(C)** oxytetracycline (10.0 μg/i.t.) on mechanical hyperalgesia of mice injected with CFA. Each circle represents the mean ± S.E.M. (n=6-7) and is representative of 2 independent sets of experiments. Statistical analyses were performed by two-way ANOVA followed by a Tukey’s test. The dashed line and number symbol indicate the range of data points where injured animals differed from the group injected intraplantarly with PBS (P<0.001).

To test whether suramin or gossypetin protect from inflammatory persistent pain in mice, we examined their effects on mechanical hyperalgesia induced by delivery of Complete Freund’s adjuvant (CFA) into the hindpaw. As shown in Figure 5, CFA induces peripheral inflammation that triggers mechanical hyperalgesia that remains significant for days when compared with either the baseline responses or the non-inflamed group (P< 0.001). Intrathecal treatment of mice with either suramin (1–10 μg/i.t., Figure 5A) or gossypetin (1–10 μg/i.t., Figure 5B), but not with the negative control compound oxytetracycline hydrochloride (10 μg/i.t., Figure 5C) or vehicle (PBS, 10 μl/i.t.) resulted in dose-dependent, rapid (15 minutes after treatment) and long-lasting anti-hyperalgesia that remained significant up to 3 hours after delivery (P < 0.001 and P<0.01, respectively for suramin and gossypetin).

**Figure 6:**
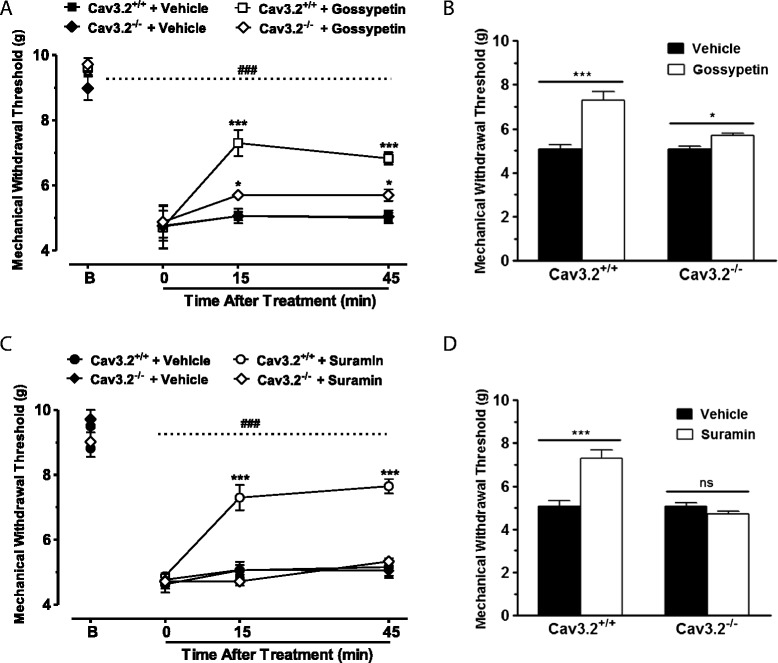
**Effect of compounds on hyperalgesia of Ca**v**3.2 null mice. (A)** Blind-analyses of the time course of mechanical hyperalgesia of CFA injected Cav3.2 null and WT mice treated with gossypetin (10.0 μg/i.t.). **(B)** Data for mechanical hyperalgesia of Cav3.2 null and WT mice when measured 15 minutes after treatment with gossypetin. **(C)** Mechanical hyperalgesia of CFA injected Cav3.2 null and WT mice treated with suramin (10.0 μg/i.t.). **(D)** Data for mechanical hyperalgesia of Cav3.2 null and WT mice when measured 15 minutes after treatment with suramin. Each circle/bar represents the mean ± S.E.M. (n=6-7) and data are representative of 2 independent sets of experiments. Statistical analyses were performed by two-way ANOVA followed by Tukey’s test. The dashed line and hashtag indicate the range of data points where injured animals differed from the sham treated group (P<0.001). Ns: non-significant.

To test if the analgesic effects of suramin and gossypetin are mediated via modulation of Cav3.2 channels, we performed a series of blind experiments in Cav3.2 null mice. In response to CFA, Cav3.2 knockout mice develop mechanical hypersensitivity that is similar to the one seen in WT animals (likely due to compensatory processes). Gossypetin (10 μg/i.t., Figure 6A, B) mediated a drastically reduced, but still statistically significant effect in Cav3.2 null mice (P<0.01), suggesting that gossypetin may have more than one molecular target *in vivo*. However, Cav3.2-null mice were completely insensitive to treatment with suramin (10 μg/i.t., Figure 6C, D), whereas this compound still produced a significant effect in WT animals when they were examined in parallel. Altogether, these data indicate that both compounds mediate their actions *in vivo* largely (and in case of suramin, exclusively) via Cav3.2 channels.

To rule out the possibility that these compounds may be direct inhibitors of Cav3.2 channels, rather than acting by uncoupling USP5 from the channel, we performed whole-cell patch-clamp experiments from tsA-201 cells transfected with cDNA encoding human Cav3.2 during which we acutely applied 10 μM suramin and gossypetin. No significant effects on current amplitude or biophysical properties of the channel were observed (7±2% inhibition (n=3) and 3±1% inhibition (n=3) of channel activity for suramin and gossypetin, respectively, which is indistinguishable from rundown), consistent with an action on the USP5-Cav3.2 channel interaction rather than direct effects on Cav3.2 channel function.

### Effects of suramin in models of acute visceral pain and diabetic neuropathy

**Figure 7:**
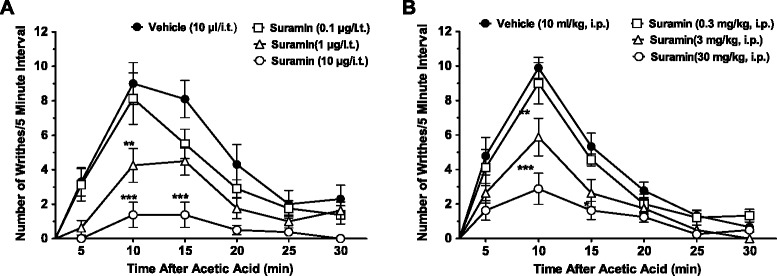
**Effects of suramin on acute visceral pain. (A)** Analyses of writhing responses of intrathecally treated mice with suramin (0.1-10.0 μg/i.t.)1 hour prior to acetic acid injections. **(B)**. Effect on intraperitoneally delivered suramin (0.3-30 mg/kg, 1 hour prior ) on writhing responses induced by acetic acid. Each circle represents the mean ± S.E.M. (n=8-10) and is representative of 2 independent sets of experiments. Statistical analyses were performed by two-way ANOVA followed by a Tukey’s test. The dashed line and number symbol indicate the range of data points where diabetic neuropathic animals differed from the non-diabetic group (P<0.001).

In light of the Tat-Cav3.2-III-IV effects described in Figure 1, it is expected that suramin should be effective in visceral pain and diabetic pain. Figures 7A and B examine the effects of suramin on acetic acid induced writhing behavior. Intrathecal (0.1 – 10 μg) or intraperitoneal (0.3 – 30 mg/kg) delivery of suramin one hour prior to acetic acid injection dose-dependently inhibited writhing behavior.

**Figure 8:**
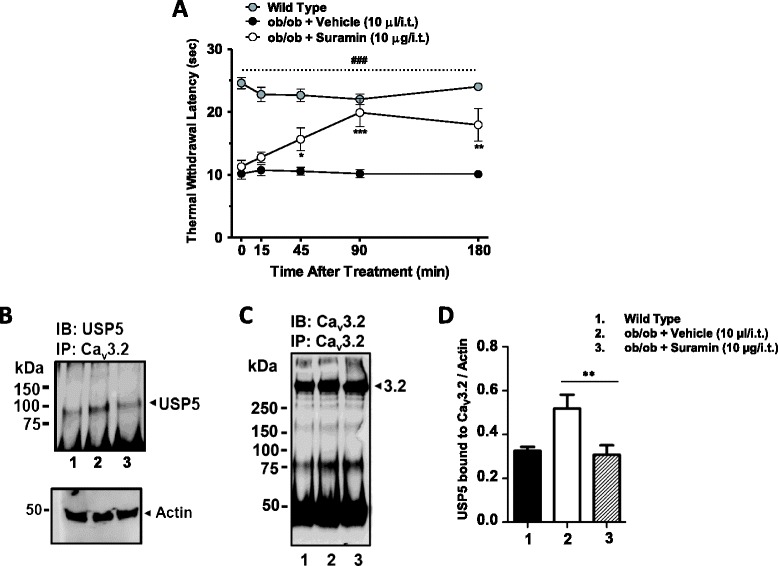
**Effects of suramin on diabetic neuropathy.** Blind analyses of time course of antihyperalgesic effect of suramin (10.0 μg/i.t.) delivered to diabetic neuropathic *(ob/ob*) and wild-type mice. The experimenter was blind to the treatment in panel **A**. Each circle/bar represents the mean ± S.E.M. (n=6) and is representative of 2 independent sets of experiments. Statistical analyses were performed by two-way ANOVA followed by a Tukey’s test. The dashed line and number symbol indicate the range of data points where diabetic neuropathic animals differed from the non-diabetic group (P<0.001). **(B)** Cav3.2 immunoprecipitates from dorsal horns from naïve mice **(lane 1)**, or from diabetic mice treated with either vehicle **(lane 2)** or suramin **(lane 3)**, were probed for USP5 and analyzed by western blot 90 minutes after i.t. treatment. A blot for actin is shown as control (bottom panel). **(C)** Cav3.2 immunoprecipitates from dorsal horns from naïve mice **(lane 1)**, or from diabetic mice treated with either vehicle **(lane 2)** or suramin **(lane 3)**, were probed for Cav3.2 channels, as control. **(D)** Quantification of USP5 bound to Cav3.2 immunoprecipitates by densitometry. Statistical analysis for mice treated with vehicle vs. mice treated with suramin was done via a Student’s t-test (^*^P <0.05).

As shown in Figure 8A, a blind experiment revealed that suramin (10 μg/i.t.) almost completely reversed thermal hypersensitivity in chronically obese mice within 90 minutes of intrathecal delivery, in agreement with the data with the Tat-Cav3.2-III-IV peptide. Next, we determined if suramin altered endogenous USP5-Cav3.2 protein interactions in the dorsal horns from diabetic and non-diabetic mice. Diabetic mice were treated with suramin (10 μg/i.t.) or vehicle and 1 hour later their dorsal horns were isolated and Cav3.2 immunoprecipitates were probed for USP5. Suramin inhibited USP5 binding to Cav3.2 channels obtained from *ob/ob* mouse dorsal horns while total Cav3.2 protein levels remained similar (Figure 8B, C). We also noticed an increase in USP5 bound to the channels from diabetic mouse dorsal horns (*ob/ob* + vehicle) when compared to wild type mouse tissue (Figure 8B, D). This likely reflects an upregulation of USP5 protein levels in neuropathic diabetic mice, which would be consistent with our previous findings in CCI and CFA animal pain models [15]. Altogether, these data fit with our observations with the Tat-Cav3.2-III-IV peptide and highlight suramin as a broadly acting analgesic in a wide range of pain conditions.

## Discussion

We recently reported that USP5-mediated deubiquitination of Cav3.2 channels in mouse dorsal root ganglia and dorsal horns is enhanced following peripheral inflammation or nerve injury, leading to increased T-type calcium currents that in turn give rise to pain hypersensitivity [15]. We showed that depletion of USP5, or its uncoupling from Cav3.2 channels via delivery of Tat peptides prevented and/or reversed the pain phenotype in models of peripheral inflammation (i.e., formalin and CFA) and peripheral nerve injury (i.e., partial sciatic nerve ligation). Here, we have extended our observation to two additional pain models – diabetic neuropathic pain and acute visceral pain. Diabetic neuropathy is one of the most prevalent complications associated with diabetes, with approximately half of type 1 and 2 diabetic patients being affected [22-24]. Diabetic neuropathy can lead to the development of chronic neuropathic pain, a debilitating condition that affects ~ 20% of diabetics and is poorly managed with current treatment regimens [25-28]. Along these lines, visceral pain is a major symptom of conditions such as inflammatory bowel disease and is challenging to manage [29]. Our observations with the Tat-Cav3.2-III-IV peptide thus suggest that targeting Cav3.2 deubiquitination by USP5 is a potential new target for therapeutic approaches to pain hypersensitivity in diabetes and pain conditions associated with inflammatory disorders of the gut.

The observation that small organic molecules can mimic the effect of the Tat peptide *in vitro* and *in vivo* is particularly exciting and it is remarkable to have identified multiple “hits” in a relatively small screen. The biflavenoid gossypetin (318 Da) and the polysulphonated naphtylurea suramin (~1.3 kDa) are vastly different in size, and yet both compounds effectively disrupted USP5 binding to the Cav3.2 III-IV linker region. Suramin is a structurally symmetric compound with two arms that each contain a sulfonated naphthalene structure. The latter is reminiscent of the hydroxylated chromene core of gossypetin, thus suggesting the possibility that the disruption of USP5-Cav3.2 interactions may involve this bicyclic aromatic structure. It will thus be interesting to conduct additional structure-activity relationship studies around the bicyclic aromatic core structure. By contrast, oxytetracycline is based on an octahydrotetracene scaffold which contains four fused benzene rings, and was inactive in both biochemical and *in vivo* assays.

To our knowledge, there is not a single report in the scientific literature that directly links the flavonoid gossypetin to pain relief, however, antinociceptive effects of crude hibiscus extract in an acetic acid writhing test in mice [30] have been reported, and there is anecdotal evidence that hibiscus tea may aid the relief of menstrual pain. In contrast, suramin has been reported to have antinociceptive effects in a formalin model of inflammatory pain [31-33] and a recent study has reported that suramin reduces mechanical hypersensitivity associated with trigeminal neuropathic pain [34]. Our data showing protection from sciatic nerve injury induced neuropathic pain fits with the latter observation. In addition, our data show that suramin protects from diabetic neuropathy, visceral pain and pain associated with persistent inflammation of the hindpaw (i.e., CFA model).

It is noteworthy that prior studies reporting analgesic effects of suramin have attributed its effects to antagonism of P2X receptors. Indeed suramin blocks P2X2/3 heteromeric receptors with an affinity in the lower micromolar range [35]. In cultured rat DRG neurons, sub micromolar concentrations of suramin have been shown to block native P2X receptors activated by 10 μM ATP [36]. This could lead one to conclude that the observed analgesic effects in our study are mediated by P2X receptor antagonism. However, our data show that the effects of suramin were completely lost in Cav3.2 null mice. This in turn indicates that suramin acts, at least in the CFA model, via Cav3.2 T-type calcium channels, rather than an action on P2X receptor. Furthermore, the activity profile of suramin mirrored that observed with the Tat-Cav3.2-III-IV peptide here and in our previous work [15], and with that of gossypetin, consistent with a common molecular mechanism of action. Only when we tested suramin in Cav3.2 null mice at 5 fold higher doses (50 μg/i.t.), we observed an additional antihyperalgesic effect with inhibition of 43 ± 16% (n=6, P<0.05), perhaps due to the antagonism of P2X receptors at higher suramin concentrations.

While suramin was initially designed to treat parasitic infections, this compound also has been tested as a potential treatment for various cancers [37]. Interestingly, in three clinical trials that examined the efficacy of suramin in the treatment of prostate cancers, patients reported reduced pain, and there was reduced opioid intake [38-40]. While these analgesic effects were not systematically investigated in a randomized clinical trial, these observations are consistent with our findings in mice and suggest potential applications for suramin as a pain therapeutic. Since both suramin and gossypetin are already approved for use in humans, these compounds could be readily entered into clinical trials for pain. Dietary supplements containing gossypetin are available and have been tested in clinical trials for efficacy in urinary tract infections [41], and hence human studies evaluating the potential beneficial effects of gossypetin in treating pain could be initiated quickly.

## Conclusions

Overall, our results indicate that disrupting the interaction between the deubiquitinase USP5 and Cav3.2 calcium channels via a small organic molecule is a promising new strategy for treating a spectrum of chronic pain states. The identification of both suramin and gossypetin as examples of such compounds, and their abilities to reverse pain hypersensitivity may potentially open an avenue for rapidly translating our findings towards clinical use.

## Methods

### Compounds and reagents

The following compounds were used in the study: Complete Freund’s Adjuvant (CFA) (Sigma Chemical Company, St. Louis, MO, USA). Suramin sodium salt (Cat# 574625, EMD Millipore) Gossypetin (Cat# G-500, Indofine Chemical Company) and oxytetracycline hydrochloride (Cat# O5875, Sigma). When compounds were delivered by the intraperitoneal (i.p.) route, a constant volume of 10 ml/kg body weight was injected. When compounds were administered intrathecally (i.t.), 10 μl were injected. Appropriate vehicle (PBS)-treated groups were assessed simultaneously. Compounds delivered to animals were dissolved in PBS.

### ELISA peptide competition assays

Recombinant human USP5 or isopeptidase T long isoform (0.5 μg) (Enzo Life Sciences) was incubated or not with 50 μg of non-biotinylated peptide (human Cav3.2_1569–1589_-III-IV) for 1 hour tumbling at room temperature (RT). Neutravidin coated plates (Piercenet) were washed with 100 μl buffer (50 mM Tris, 150 mM NaCl+0.05% Tween-20) prior to addition of peptides. Then 5 μg of the Cav3.2 biotinylated peptide III-IV linker (human Cav3.2_1556–1602_-III-IV) was added to the 96-well plate with the preincubated complex of recombinant hUSP5 long (0.5 μg/ 1.25 μl) + non-biotinylated peptide for 1 hr incubation at RT. Wells were washed three times with 100 μl Tris-NaCl-150, shaking at RT for 10 minutes each, and 100 μl blocking buffer-TBS (Piercenet) was added to wells. Plates were immediately emptied. 100 μl of blocking buffer was added and samples were incubated for 1 hr at RT while shaking. Plates were inverted to clear the wells. Plates were then dried three times on a stack of paper towels. 100 μl rabbit primary anti-USP5 antibody (1:1,000 dilution; ProteinTech Group, Inc.) was added to each well and incubated for 1 hr at RT. Plates were inverted to empty wells and washed three times with 200 μl of wash buffer (50 mM Tris, 150 mM NaCl + 0.05%Tween-20) for 10 minutes each on a shaking platform. Plates were dried three times as before. Samples were incubated with anti-rabbit HRPconjugated secondary antibody (Jackson Immunoresearch Labs; 1:10,000 dilution) for 45 min at RT. Plates were inverted and dried as before. 100 μl of QuantaBlu WS (Pierce) (9 parts substrate solution + 1 part stable peroxide) was added to each well and incubated for 1.5-90 min at RT. Peroxidase activity was stopped by adding 100 μl of QuantaBlu Stop Solution (Pierce). The excitation and emission maxima for QuantaBlu Fluorogenic Peroxidase Substrate were 325 nm and 440 nm, respectively. A VarioSkan microplate reader (Thermo Scientific) was used to take relative fluorescence unit (RFU) readings (Ex/Em: 325/440 nm).

### Cav3.2/USP5 ELISA screening assay

Black high-binding 384-well plates (Corning) were incubated (overnight, 4°C) with 4 μg/ml Neutravidin (Thermo Scientific) in 40 μl PBS with 0.05% azide which was added using a WellMate dispenser (Matrix Technologies). The plates were washed 3 times with 90 μl Tris buffer (50 mM Tris, 150 mM NaCL, pH 7.5) with a plate washer (BioTek), after which plates were incubated with 80 μl blocking buffer (Tris buffer plus 1% BSA (Sigma);1 hr, RT). Plates were then washed 3 times with Tris buffer and the WellMate used to add 25 μl Tris buffer per well. A 5,000 compound library from CDRD containing known drugs and bioactives, known as the KD2 library, was pinned using a PlateMate Plus machine (Matrix Technologies) and a FP3 pintool, to give a final compound concentration of 6.3 μM per well. The biotinylated Cav3.2 III-IV linker peptide (0.0039 μg/μl Genemed synthesis Inc.) was added in 15 μl Tris buffer (1 hr, RT). This was followed by 15 μl of 0.00078 μg/μl USP5 protein (Enzo Life Sciences) in Tris buffer added to all wells apart from the negative control wells (1 hr, RT). Wells were washed three times with wash buffer (Tris buffer plus 0.05% Tween (Fisher)), followed by the addition of anti-USP5 primary antibody (Proteintech Group) in 30 μl blocking buffer (1:5000 dilution, 1 hr, RT) per well. Wells were subsequently washed 3 times with wash buffer, after which anti-rabbit HRP-conjugated secondary antibody (Jackson Immunoresearch Labs) was added in 30 μl blocking buffer, (1:15,000 dilution, 1 hr, RT) per well. After washing three times, 30 μl QuantaBlu substrate (Pierce) was added per well (5 min, RT) followed by 30 μl QuantaBlu stop solution. A VarioSkan microplate reader (Thermo Scientific) was used to take RFU readings (Ex/Em: 325/440 nm). The raw data obtained were converted to % inhibition and Z’ and signal to noise (S/N) values were calculated.

### Co-immunoprecipitation assays

Mouse brain tissue was lysed in a modified RIPA buffer (in mM; 50 Tris, 100 NaCl, 0.2% (v/v) Triton X-100, 0.2% (v/v) NP-40, 10 EDTA + protease inhibitor cocktail, pH 7.5) that was used to co-immunoprecipitate Cav3.2 channels with USP5 protein. Lysates from mouse dorsal horn tissue were prepared by sonicating samples at 60% pulse for 10 seconds and by centrifugation at 13,000 rpm for 15 minutes at 4°C. Supernatants were transferred to new tubes and solubilized proteins were incubated with 50 μl of Protein G/A beads (Piercenet) and 2 μg of anti-Cav3.2 (H-300, Santa Cruz Biotechnologies, Inc.,) antibody overnight while tumbling at 4°C. Total inputs were taken from whole cell samples representing 4% of total protein and probed for actin. Co-immunoprecipitates were washed twice with (mM) 150 NaCl 50 Tris pH 7.5 buffer, beads were aspirated to dryness. Laemmli buffer was added and samples were incubated at 96°C for 7 minutes. Eluted samples were loaded on a 10% Tris-glycine gel and resolved using SDS-PAGE. Samples were transferred to 0.45 mm polyvinylidenedifluoride (PDVF) membranes (Millipore) and western blot analysis was performed using anti-actin (Sigma), and anti-USP5 (ProteinTech Group, Inc.) antibodies. Western blot quantification was performed using densitometry analysis (Quantity One-BioRad software). Student’s t-tests for unpaired data were performed to determine statistical significance.

### Animals

Experiments were performed after approval of an animal protocol by the Institutional Animal Care and Use Committee and all efforts were made to minimize animal suffering according to the policies and recommendations of the International Association for the Study of Pain. In this study we used male C57BL/6 J (wild-type), *Cacna1h* (Cav3.2 null, background C57BL/6 J) mice (*mus musculus*, 22-28 g, 8 weeks old) or morbidly obese (*ob/ob,* background C57BL/6 J, 14–17 weeks old) purchased from the Jackson Laboratory. Animals were housed at a maximum of five per cage (30 × 20 × 15 cm) and with water and food *ad libitum.* They were kept in controlled temperature of 23 ± 1°C on a 12 h light/dark cycles (lights on at 7:00 a.m.) and all experiments were performed between 10 am and 3 pm. Different cohorts of mice were used for each test. The observer was blind to the experimental conditions in the experiment examining the effect of the Tat-III-IV-linker peptide and suramin when delivered intrathecal to mice on acetic acid model. Diabetic (*ob/ob*) mice and their age-matched wild-type counterparts (C57BL/6) were studied for thermal hyperalgesia between the ages of 14 and 17 weeks. Upon their arrival at the age of 6 weeks, mouse body weights and blood glucose levels were monitored monthly by analyzing tail blood samples by using a One Touch-Verio glucometer (Johnson & Johnson, New Brunswick-NJ). Neither suramin nor the Tat-III-IV-linker peptide altered glucose levels of diabetic animals. Obese mice were tested for thermal hyperalgesia due to their excessive bodyweight that compromises mechanical threshold measurements using the digital aesthesiometer. The experimenter was blind to the experimental conditions in the experiment evaluating the antinocicpetive action of the Tat-disruptor peptide on acetic acid test (Figure 1A, B); the antihyperlagesic action of the Tat-disruptor peptide on diabetic neuropathy (Figure 8A), and the effect of suramin and gossypetin on chronic inflammatory pain (CFA model) in either wild-type or Cav3.2 null mice (Figure 6).

### Persistent inflammatory pain induced by CFA

To induce mechanical hyperalgesia produced by peripheral inflammation, 20 μl of Complete Freund’s Adjuvant (CFA) was injected subcutaneously in the plantar surface of the right hindpaw (i.pl.) [42]. Sham groups received 20 μl of PBS in the ipsilateral paw. Animals were treated with either suramin (1–10 μg/i.t.), gossypetin (1–10 μg/i.t.), vehicle (10 μl/i.t.) or oxytetracycline hydrochloride (10 μg/i.t., also used as negative control), 3 days following CFA injection and their mechanical withdrawal threshold were subsequently tested.

### Mononeuropathy caused by partial sciatic nerve injury

In order to produce neuropathic pain in mice, a partial ligation of the sciatic nerve was performed according to [43]. Briefly, under isoflurane anesthesia and aseptic conditions the right sciatic nerve was exposed at high-thigh level and a 6–0 silk suture was inserted into the nerve and tightly ligated so that the dorsal 1/3-1/2 of the nerve thickness was trapped in the ligature and the wound was closed with 4–0 silk suture. In all mice the left (contralateral) leg and sciatic nerve were untouched. In sham operated mice the nerve was left intact. Fourteen days later the operated mice received suramin (10 μg/i.t.), gossypetin (10 μg/i.t.), or vehicle (10 μl/i.t. of PBS). Measurements of mechanical withdrawal thresholds were taken as described below.

### Acute visceral pain

To produce acute visceral pain, mice were injected with 0.45 mL of a 0.9% v/v solution of acetic acid in PBS intraperitoneally. This results in a release of pro-nociceptive inflammatory mediators from resident mast cells and macrophages [44] and a pain response characterized by writhing, full extension and contraction of the abdomen [45,46]. One hour prior to acetic acid injection, mice were treated intrathecally with the Tat-3.2-III-IV-linker (10 μg/i.t.), the Tat-3.2-CT (control, 10 μg/i.t.) peptides, with suramin either intrathecally (0.1-10 μg/i.t.) or intraperitoneally (0.3-30 mg/kg, i.p.). After acetic acid administration, animals were immediately placed in individual observation chambers and numbers of writhes were counted in 5-minute intervals for 30 minutes after acetic acid injection.

### Mechanical hyperalgesia

For evaluation of mechanical hyperalgesia we used the digital plantar aesthesiometer (DPA, UgoBasile, Varese, Italy) according to our previous publication [15]. Mice were placed individually in a small enclosed testing arena (20 cm × 18.5 cm × 13 cm, length × width × height) on top of a grid floor while they acclimated in the experimental room for a period of at least 90 minutes before the measurements. The aesthesiometer was positioned underneath the animal with the filament directly under the plantar surface of the ipsilateral hind paw. To verify the time-dependence effect of suramin and gossypetin, mechanical withdrawal thresholds were determined at 1 day prior to CFA injection (Baseline), and 3 days after CFA injection at 0, 15, 45, 90 and 180 minutes after treatment of mice with either suramin, gossypetin or oxytetracycline. Each paw was tested three times per session.

### Thermal hyperalgesia

Thermal hyperalgesia was examined on diabetic and non-diabetic (*ob/ob*) mice by measuring the latency to withdrawal of right hind paws on a focused beam of radiant heat (IR=30) of a Plantar Test apparatus (UgoBasile, Varese, Italy). Animals were placed individually in a small enclosed testing arena (20 cm × 18.5 cm × 13 cm, length × width × height) on top of a wire mesh floor. Mice were allowed to acclimate for a period of at least 90 minutes. The device was positioned beneath the animal, so that the radiant heat was directly under the plantar surface of the ipsilateral hind paw. Three trials for each mouse were performed. The apparatus was set at a cut-off time of 30 s to avoid tissue damage. Thermal hyperalgesia was evaluated immediately prior to the treatments (Time 0) and 15, 45, 90 and 180 minutes after treatment when mice were between 14–17 weeks old.

### Intrathecal drug treatment

Intrathecal injections were perform in fully conscious mice as previously described [47]. Briefly, mice were manually restrained, the dorsal fur of each mouse was shaved, the spinal column was arched, and a 30-gauge needle attached in a PE20 Polyethylene tube to a 25-μl Hamilton microsyringe (Hamilton, Birmingham, UK) was inserted into the subdural space between the L_4_ and L_5_ vertebrae. Accurate positioning of the needle tip was confirmed by a characteristic tail-flick response of animal when the needle if correctly positioned. Intrathecal injections of 10 μl were delivered over a period of 5 seconds.

### Statistical analysis

For biochemical results data are presented as mean ± S.E.M. and statistical significance was determined using Student’s *t*-test unless stated otherwise: ^*^P <0.05; ^**^P <0.01; ^***^P <0.001; NS = statistically not different. For behavioral experiments, data are presented as means ± SEM and evaluated by one-way, two-way or three-way analysis of variance (ANOVA) followed by a Tukey’s test. A value of P < 0.05 was considered to be significant. (^*^P <0.05; ^**^P <0.01; ^***^P <0.001; NS = not different).

## Additional file

Additional file 1: Figure S1. Control experiments for acetic acid test. Writhing behavior in response to intraperitoneal injection of PBS, or acetic acid in the presence of either vehicle or the anti-inflammatory Diacerein. Note that PBS injection does not result in writhing behavior, whereas acetic acid produces writhes that can be blocked by Diacerein. Each bar includes data from five mice.
