# Supplementary material for: Small organic molecule disruptors of Cav3.2 - USP5 interactions reverse inflammatory and neuropathic pain
Source: Mol Pain. 2015 Mar 14;11:12. doi: 10.1186/s12990-015-0011-8 (PMC4364099; doi:10.1186/s12990-015-0011-8)
Supplement: Additional file 1: Figure S1. — Control experiments for acetic acid test. Writhing behavior in response to intraperitoneal injection of PBS, or acetic acid in the presence of either vehicle or the anti-inflammatory Diacerein. Note that PBS injection does not result in writhing behavior, whereas acetic acid produces writhes that can be blocked by Diacerein. Each bar includes data from five mice. [file 12990_2015_11_MOESM1_ESM.pdf]

Supplementary Data Figure 1. Control experiments for acetic acid test

Writhing behavior in response to intraperitoneal injection of PBS, or acetic acid in the presence of either vehicle or the anti-inflammatory Diacerin. Note that PBS injection does not result in writhing behavior, whereas acetic acid produces writhes that can be blocked by Diacerin. Each bar includes data from five mice.

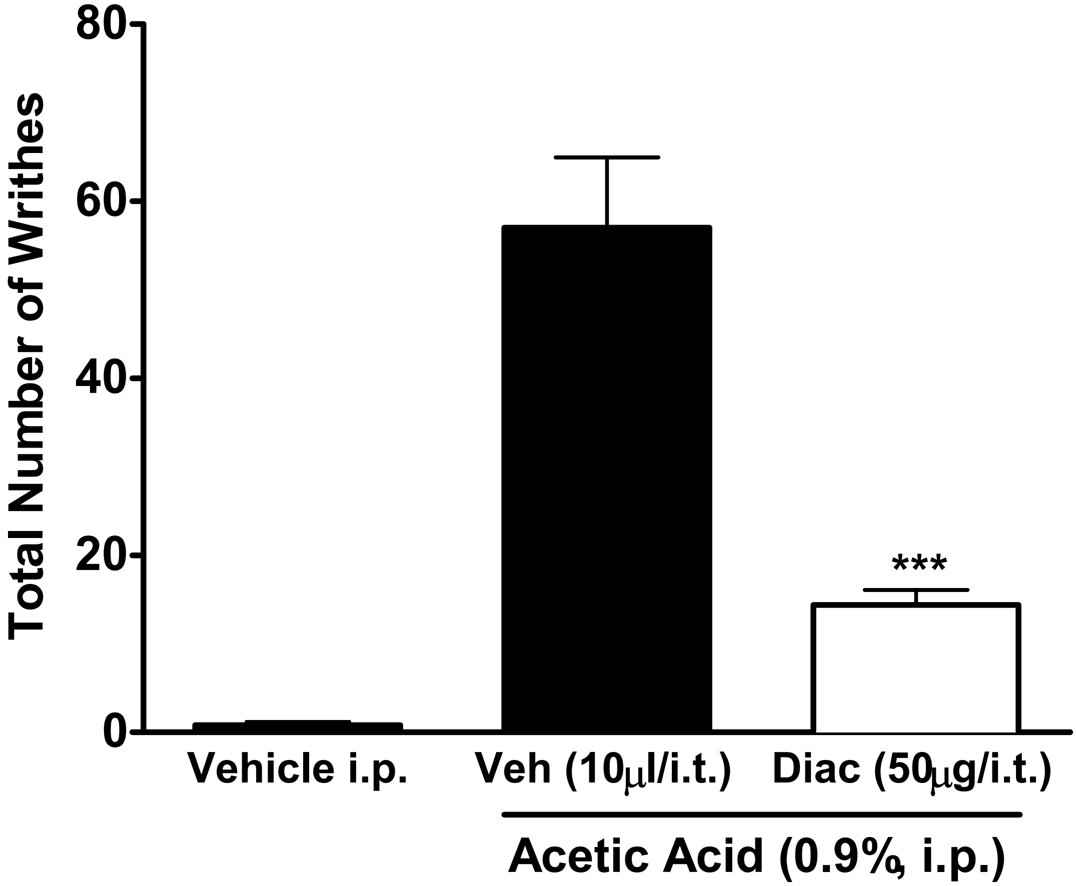
Total Number of Writhes
80
60
40
20
0
***
Vehicle i.p.
Veh (10μl/i.t.)
Diac (50μg/i.t.)
Acetic Acid (0.9%, i.p.)
